# The Objections of the Profession to Trade Unions

**Published:** 1913-08-09

**Authors:** 


### THE NATIONAL MEDICAL GUILD.

### The Objections of the Profession to Trade Unions.

Last week we devoted our space to an examination of the position created by the vote of the representatives at the annual meeting at Brighton. This week we propose to deal with the commoner objections to trade unionism and to an attempt to show the superior strength of a trade union for fighting purposes.

When one associates oneself with any movement one must almost inevitably be touched by all the odium that the movement or its adherents have created in the past. That is a legacy from which there is no escape, and which must be put up with until sufficient time has elapsed to live it down. So it is with those of us who are unionists. In the past much has been done by trade unions that is regrettable, and it is natural that the opponents of the movement, anxious to find arguments against it, will point the finger of scorn at the organisers, and jeeringly inquire if the medical trade union is going to follow the same path. Ours the task to show them that trade unionism can, in its purest form, be a beautiful thing, and by our action to silence criticism.

### Beginnings of Mutual Aid.

To do this one must go back to the primary inception of mutual protection. This banding of men together was always the result of one thing—viz., oppression; and the first persons that we find joining hands for mutual support are the tradesmen of the City of London, whose Guilds are world famous, although the reason for their existence has long since departed. Amongst these Guilds were the Barber surgeons, who did not disdain to avail themselves of what protection they could. As time went on it was recognised that these bodies were collections of law-abiding citizens, banded together for lawful purposes, for mutual help, and for the aid of their poorer brethren and their dependants, and they came to hold positions of honour in the realm, and incidentally to collect great wealth. From these Guilds of masters and master workmen the workmen of another century took a lesson, and they, too, tried the results of combination. What these results have been are well known to everyone, and the power that a union has over a man's work and wages is recognised. Few will be found who would unhesitatingly approve of every action of trade unions in the last twenty years, but that is no reason why the National Medical Guild should be compelled to shoulder the sins of their predecessors and be damned in advance because it might in the future commit the same actions.

### The Strike Argument.

One of the chief arguments used against us is that we stand for a strike. This matter of the power of unions to order strikes is one that needs very careful discussion. In the first place it is usually said that the only power of a trade union is in its ability to order a strike at any moment that it will. This is one of those half truths that have been uttered so frequently lately that the unthinking have come to accept it as an axiom. Let us examine it a little more carefully. In the first place, whilst it may be admitted that the strike is the last weapon in a dispute, yet it would be interesting to know how many trade disputes have been settled in the last twenty years without the extreme method of the strike being declared. Any diligent reader of the daily paper will be able to tell of dozens of incidents; but it would be far more interesting to know how many of these disputes would have been settled satisfactorily (from the men's point of view) if the negotiations had been carried out by their leaders without the power of calling out their men if their demands were not listened to. It is the potential power of this weapon that we are continually urging upon the profession. It is a sword that might rust in its scabbard for fifty years, what time numberless small disputes were settled without unsheathing it; nevertheless it is a power in these small disputes that would help considerably in the settlement of them. What compelled the Chancellor of the Exchequer last autumn to raise the remuneration of the profession to nearly double the original offer? Did not the fact that its members had handed in their notices to terminate their contract appointments, in other words, to strike, cause this sudden generosity? The only concession that the British Medical Association wrung from an unwilling Government was at the point of a weapon that the Association now pretends to despise. Further, if the medical profession as a body in the future decided to strike it would not be a strike in the commonly accepted meaning of the term, that is to say, medicine would not cease to be practised, but the men would cease practice on the terms offered. This is a vastly different thing to stopping all work—a position that a great profession would never adopt—and yet there are men who still believe that a strike of medical men is equivalent to the "down tools" of the workman. Properly understood, the power to strike is, in the hands of a sane profession, a great potential weapon. Properly and judiciously used it could restore to the profession some of the prestige that they have lost in the late débâcle, and could place them in a position in which they could feel themselves free from any attacks in the future.

### What is Intimidation?

The next objection that was raised at Brighton was that the chief power of trade unions lay in intimidation. This may be partly true, but what is intimidation. It is the threat to take away or to withhold something from a person who will not take the course that one wishes him to. If one accepts that definition then nearly all our actions savour of intimidation. If one obtains the signacare of a friend to a pledge on the grounds that he is a friend, that is intimidation. That man signs because he is afraid if he does not his friend will be his friend no more. This is a different kind of intimidation to the profuse use of brick ends of which we sometimes read, but it is nevertheless intimidation. We would like to ask the British Medical Association one question. When the Warning Notices are published in the "Journal," and they succeed in persuading intending applicants to refrain from sending in their applications, on what grounds do they imagine does the applicant so restrain himself? Surely because he does not wish to act in opposition to the declared wishes of the profession in the neighbourhood. If so, then that must become a species of intimidation of a very mild order. We have written this in no spirit of criticism of the excellent work done by the "Journal" in its Warning Notices, but simply to show the members that they are using a mild form of the same thing that they so vigorously condemn in other bodies. That the National Medical Guild will ever go about the country using missiles as arguments is, of course, ridiculous.

### The Privileges of Trade Unions.

And now we think that the time has come to answer a perfectly reasonable question that is often put to us, and that is, How can a trade union do all these things better than our existing organisations? The answer is contained in a few words. Because a trade union has taken to itself those special immunities and privileges that are granted to it in virtue of its registration. These few words need considerable elaboration.

In the matter that we had under discussion last week—the safety of the funds of the union—we showed how dangerous it was for any body other than a trade union to raise funds which they proposed to use in the restraint of trade, and how liable those funds were to attachment by the very persons whom one was fighting at the moment that they were required, and at the risk of being considered reiterative we would again call attention to this important matter. No man would go to war with his ammunition in the hands of the enemy, and yet that is the position of a body that proposes to raise funds without seeking that immunity that a trade union alone can give. If this were the only reason for our existence we should feel more than justified, but there are others.

A moment ago, in speaking of intimidation, we mentioned the excellent work of the British Medical Association in their Warning Notices. Is it a matter of common knowledge that those Notices are only inserted after the most careful consideration has been given to them, and even then their legality is slightly doubtful? If those same Notices were published by a trade union their legality would be beyond question, as they are issued when a trade dispute is pending, and a registered trade union is exempt from any civil proceedings in such a matter. Likewise its officials and members have immunity from tortious acts committed in the pursuit of any such dispute, a very valuable protection, as the perusal of the legal costs of the Association will testify.

### The Guild's Scheme of Retiring Pensions.

Again, in the matter of calling a strike, it is quite true that the Association is quite able to call upon its members to refrain from certain work until the terms and conditions are agreed by the whole profession. It did so once, and we all know the result. The causes that led to that result are a subject for the historian, and may be left to him; but the hold that the Guild hopes to get upon its members will be greater than any Association can ever hope to obtain. For example, in the first place no man deliberately goes out of his way to be turned out of his professional organisation; indeed, the threat of expulsion was sufficient to bring one recalcitrant member to his senses in a small dispute that we settled. That power of expulsion is held in a much greater degree by a trade union than by any other body, and the Guild will not hesitate to use it when required, and, what is of more importance, to advertise the fact of the member's expulsion, another privilege that trade unions enjoy.

Secondly, it is the hope of the Guild that they will be able to establish and place before their members a scheme of retiring pensions at co-operative rates—a scheme of life insurance at lower rates than existing companies can grant. Something has been done in that direction, and already it is cheaper to insure anything through the Guild than any private company.

Finally, it is the desire of the Guild to establish in all places a scheme of medical service for all working classes on lines similar to that in Leicester. A scheme is now being put into operation in one London borough under our ægis. If the result be successful we hope to extend it to the country. These may be ideals, but they are both sane and possible if the profession will help us, and by helping us help themselves. What a hold on the profession such a body could have. With his life, and his pension insured through the Guild, his place in the public medical service dependent upon his retaining his membership in the Guild, with all his brother practitioners members of the same brotherhood, the position of the profession would be unassailable, and from a collection of individualistic units we should become a solid and united body. Will that day come? The answer is in the hands of the profession itself.

